# Use of Proton Pump Inhibitors in Hungary: Mixed-Method Study to Reveal Scale and Characteristics

**DOI:** 10.3389/fphar.2020.552102

**Published:** 2020-09-08

**Authors:** Mária Matuz, Ria Benkő, Zsófia Engi, Krisztina Schváb, Péter Doró, Réka Viola, Mária Szabó, Gyöngyvér Soós

**Affiliations:** ^1^ Department of Clinical Pharmacy, Faculty of Pharmacy, University of Szeged, Szeged, Hungary; ^2^ Department of Surgery, Albert Szent-Györgyi Health Center, University of Szeged, Szeged, Hungary

**Keywords:** proton pump inhibitors, proton pump inhibitors exposure, proton pump inhibitors dose, proton pump inhibitors therapy duration, nationwide data, patient-level survey

## Abstract

**Background:**

Due to their efficacy and tolerability, utilization of proton pump inhibitors (PPI) has significantly increased worldwide. Parallel to the clinical benefits, potential long-term side effects have been observed, which, along with increasing medical expenses and potential drug interactions, justifies the analysis of the trends of utilization.

**Objective:**

The aim of the present study was to show the level, pattern, and characteristics of PPI use.

**Methods:**

We assessed the nationwide use of proton pump inhibitors in ambulatory care based on aggregated utilization data from the National Health Insurance database. The annual PPI utilization was expressed as the number of packages and as number of DDDs per 1,000 inhabitants and per year. For 2018, we estimated PPI exposure as the number of packages and as the number of DDDs per user per year. The annual reimbursement costs of proton pump inhibitors were also calculated. Moreover, three patient-level surveys were carried out in non-gastroenterological inpatient hospital departments to reveal characteristics of proton pump inhibitor use, namely dose, duration, and indication.

**Results:**

The PPI utilisation increased from 5867.8 thousand to 7124.9 thousand packages and from 41.9 to 50.4 DDD per 1,000 inhabitants and per day between 2014 and 2018. Nationwide data showed that 14% of the adult population was exposed to proton pump inhibitors in 2018, while among hospitalized patients, the prevalence of proton pump inhibitor use was between 44.5% and 54.1%. Pantoprazole was the most frequently used active ingredient, both in the nationwide data and in the patient-level surveys. In the patient-level survey in majority of patients (71.5%–80.0%) proton pump inhibitors were prescribed for prophylaxis. Many inpatients (29.4%–36.9%) used 80 mg pantoprazole per day. The average number of PPI packages per user was 6.5 in 2018 in the nationwide data. The duration of PPI therapy was typically between 1 and 5 years in the patient-level surveys and nearly 20% of the inpatients had been taking proton pump inhibitors for more than 5 years.

**Conclusions:**

Our data suggests that Hungarian patients receive proton pump inhibitors in high doses and for a long time. Use of proton pump inhibitors beyond their recommended indications was also found.

## Introduction

In the 1990s, widespread use of proton pump inhibitors (PPIs) radically changed the treatment of acute and chronic gastroenterological diseases caused by hyperacidity (Garner et al., 1996; Savarino et al., 2018a). Different PPI products have an equivalent mechanism of action and a similar clinical efficacy (Strand et al., 2017). Due to their excellent efficacy, tolerability and positive adverse event profile, PPI utilization has significantly increased all over the world (Hollingworth et al., 2010; Tett et al., 2013; Lanas, 2016; Ying et al., 2019). Besides official, evidence-based use of PPIs, less understandable and justifiable prescriptions have also occurred (Savarino et al., 2017; Savarino et al., 2018a). More than 10 years ago, additional costs resulting from PPI overuse were estimated to be 2 billion pounds worldwide (Forgacs and Loganayagam, 2008).

Parallel to the clinical benefits of long-term PPI therapy in some clinical conditions (e.g. severe erosive esophagitis, Barett’s esophagus), potential side effects have also been observed, especially among the elderly (Maleth and Hegyi, 2013; Freedberg et al., 2017; Maes et al., 2017; Yu et al., 2017; Haastrup et al., 2018; Igaz et al., 2018; Devitt et al., 2019; Lanas-Gimeno et al., 2019). Some of these are controversial and based on extremely loose associations (e.g. dementia, chronic nephropathy), while others (e.g. enteral infections, micronutrient deficiency) have been confirmed by sound evidence (Bajor, 2017; Strand et al., 2017; Ayele et al., 2018; Schubert, 2018; Schubert, 2019).

Potential long-term side effects, increasing medical expenses, and potential drug interactions (Hu et al., 2018) justify the recognition and analysis of the trends and characteristics of PPI utilization. Utilization of PPIs has been analyzed in many countries (Pillans et al., 2000; Chia et al., 2014; Larsen et al., 2014; Kelly et al., 2015; Meli et al., 2015; Lodato et al., 2016; Pottegard et al., 2016; Pujal Herranz, 2016; Juntunen et al., 2017; Del Giorno et al., 2018; Halfdanarson et al., 2018; Luo et al., 2018; Ying et al., 2019), but no data from Hungary has been published yet, as emphasized in the study of Igaz et al. (2018). The aim of the present study was to show the level, pattern, and characteristics of PPI use, using mixed methods.

## Methods

### National Data

The source of annual national drug utilization data was the public, aggregated report of the National Health Insurance Fund of Hungary (Hungarian acronym: NEAK) (2014–2018). This report contains information on all dispensed and reimbursed prescription drugs in Hungary. As NEAK is the sole and mandatory health insurance agency in Hungary, it covers 100% of the population. Over the counter dispensations (including some PPI products), and non-reimbursed drugs are not included in the dataset.

The reimbursement rate is 55% for all PPI substances, regardless of the indication and the prescriber. Only the products with the highest price (usually the originator) are excluded from the reimbursement status. Currently, there are eleven PPI products that are available without prescription (nine products containing 20 mg pantoprazole and two products containing 20 mg esomeprazole), all the other PPI products (N=74) are prescription-only. General practitioners can initiate and prescribe PPI therapy with a similar reimbursement rate as gastroenterologists.

PPIs were classified using the WHO ATC [World Health Organisation (WHO), 2020] methodology (version 2020). PPI use was expressed as the number of annual packages and as WHO defined daily doses (DDD, version 2020) standardized for the population (i.e. DDD per 1,000 inhabitants and per day). Furthermore, regarding certain PPI active ingredients (ATC subgroup A02BC), for the year 2018, we determined the average number of packages per user per year and the average number of DDDs per user per year. The public drug utilization report of NEAK includes reimbursement costs. Summarizing these numbers, we calculated the annual reimbursement cost of PPIs.

### Patient-Level Surveys

Three patient-level cross-sectional surveys were carried out in inpatient hospital departments. For Surveys 1 and 2, all hospital pharmacists were invited from hospitals (N=12) where daily dose drug dispensation was supervised by a pharmacist and medical reconciliation practice was in place in 2016. This is approximately 10% of all Hungarian hospitals. Out of the 12 hospitals, 10 agreed to take part in the survey. To enable generaliability the surveys were conducted in adult departments (N=29) of these hospitals with a non-gastroenterological profile, to exclude units where acid-related diseases are specifically treated. Intensive care units and psychiatry units were not included as communication with the patient is difficult/not possible that may introduce response bias. The most important characteristics of the surveys are shown in **Table 1**. The different surveys consecutively followed each other, and revealed progressively more characteristics of PPI use (e.g. Survey 1 did not assess duration of PPI use, but Surveys 2 and 3 did). The precise overall medication regimen was recorded in Survey 3. All adult (age 18 years or above), cooperative inpatients on the specific study days were included in the surveys to avoid selection bias. On the specific study days, the responsible ward pharmacist recorded medication use from medical records (that were filled in by the pharmacist during the admission) and consulted the patients about PPI indication, therapy duration, etc. In Survey 3, the initiation of PPI therapy was also asked from the patient (GP or specialist).

**Table 1:** Methodological characteristics of patient-level Surveys 1, 2, and 3.

	**Survey 1**			**Survey 2**	**Survey 3**
**Survey Date(s)**	27 July 2016			25 January 2017	April 2017–March 2018 On 12 appointed days (1 day per 3–4 weeks)
**Survey Design**	point prevalence				point prevalence (on multiple days)
**Survey Site**	29 wards from 10 hospitals surgical, trauma, neurological, or dermatological wards (10 outside the capital; 19 in the capital)				vascular surgery ward
** Dosing of PPI therapy**	recorded		recorded		recorded
** Indication of PPI therapy**	recorded		recorded		recorded
** Duration of PPI therapy**	not recorded		recorded		recorded
** Initiation of PPI use**	not recorded		only hospital initiation		recorded
** De-prescribing attempt**	not recorded		not recorded		recorded
** Total number of the patient’s chronic medications**	not recorded	recorded			recorded

According to sample size calculations, at least 300–400 responses were required to estimate the occurance of non-rare events, such as PPI use. Due to further stratification of data we sought for a higher sample size in the multicenter surveys (Surveys 1 and 2).

The data sheet for anonymous data collection contained demographic data and characteristics (active ingredient, daily dose, etc.) of PPI treatment. In all surveys, data were obtained during the medication reconciliation process. Polypharmacy was defined as taking five or more concomitant medications chronically (Duerden et al., 2013). Descriptive statistics were used with measures of central tendency (mean) and spread of the distribution (standard deviations minimum-maximum range). Data processing and evaluation were carried out using MS Office and R.3.5.1. software.

## Results

### National Data

In 2018, a total of 7.12 million PPI packages were prescribed and dispensed, which corresponds to 730 packages per 1000 inhabitants, while in 2014, these numbers were under 5.87 million packages and 600 packages per 1000 inhabitants. Expressing the utilisation in DDD per 1,000 inhabitants and per day, it increased from 41.9 to 50.4 (see **Table 2B**). Over the last 5 years, PPI utilization has shown an upward trend, which is mostly due to an increase in pantoprazole use (**Table 2A**). Assuming that no active agent switch occurred, nearly 1.1 million Hungarian inhabitants were exposed to PPIs. This represents 14% of the adult population aged 18 years or above.

**Table 2A:** Utilization of certain proton pump inhibitors (PPIs’) over the last 5 years in Hungary (organized in descending order based on the data from 2018)—number of packages.

ATC	Active Ingredient	1,000 packages *(share in %)*					trend
		2014	2015	2016	2017	2018	
A02BC02	pantoprazole	3171.74	3842.92	4144.39	4444.86	4603	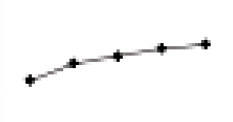
		*(54.05)*	*(58.22)*	*(60.68)*	*(62.21)*	*(64.60)*	
A02BC05	esomeprazole	1101.76	1210.43	1241.44	1307.98	1282.82	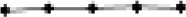
		*(18.78)*	*(18.34)*	*(18.18)*	*(18.31)*	*(18.00)*	
A02BC04	rabeprazole	768.73	742.15	696.3	673.08	596.48	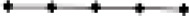
		*(13.10)*	*(11.24)*	*(10.19)*	*(9.42)*	*(8.37)*	
A02BC03	lansoprazole	630.61	586.23	538.59	495.21	417.06	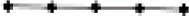
		*(10.75)*	*(8.88)*	*(7.89)*	*(6.93)*	*(5.85)*	
A02BC01	omeprazole	194.97	218.66	209.74	223.58	225.52	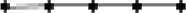
		*(3.32)*	*(3.31)*	*(3.07)*	*(3.13)*	*(3.17)*	
All PPIs (A02AB)		5867.8	6600.4	6830.47	7144.72	7124.88	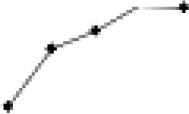
		*(100.00)*	*(100.00)*	*(100.00)*	*(100.00)*	*(100.00)*	

**Table 2B:** Utilization of certain proton pump inhibitors (PPIs’) over the last 5 years in Hungary (organized in descending order based on the data from 2018)—defined daily dose (DDD) per 1,000 inhabitants and per day.

ATC	Active Ingredient	DDD per 1,000 inhabitants and per day *(share in %)*					trend
		2014	2015	2016	2017	2018	
A02BC02	pantoprazole	21.06	25.78	28.03	29.36	30.04	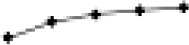
		*(50.27)*	*(54.41)*	*(56.87)*	*(57.62)*	*(59.64)*	
A02BC05	esomeprazole	9.33	10.33	10.60	11.21	11.04	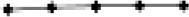
		*(22.28)*	*(21.80)*	*(21.5)*	*(21.99)*	*(21.91)*	
A02BC04	rabeprazole	5.54	5.38	5.12	5.00	4.42	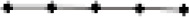
		*(13.22)*	*(11.36)*	*(10.40)*	*(9.82)*	*(8.77)*	
A02BC03	lansoprazole	4.36	4.08	3.78	3.51	2.97	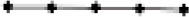
		*(10.41)*	*(8.61)*	*(7.67)*	*(6.88)*	*(5.89)*	
A02BC01	omeprazole	1.60	1.81	1.75	1.88	1.91	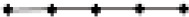
		*(3.82)*	*(3.82)*	*(3.55)*	*(3.69)*	*(3.79)*	
All PPIs (A02AB)		41.88	47.37	49.29	50.96	50.37	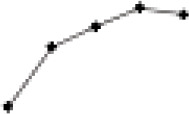
		*(100.00)*	*(100.00)*	*(100.00)*	*(100.00)*	*(100.00)*	

Every year (2014–2018), more than half of the used PPIs contained pantoprazole as the active ingredient. In 2018, more than 663,000 inhabitants redeemed at least one package of a pantoprazole product (**Table 3**), which means that 7% of the Hungarian population (including children) was exposed to pantoprazole. Considering the annual number of packages, the average number of packages per user in case of pantoprazole products was almost 7 (6.9) in 2018. Since in most cases, one package equals 1 month of PPI use, this suggests average long-term PPI therapy of up to 6 to 8 months.

**Table 3:** Annual proton pump inhibitor (PPI) use in packages and in DDDs in 2018, number of proton pump inhibitor users of certain active ingredients, and sales standardized by number of users (based on the public database of the National Health Insurance Fund of Hungary).

ATC	Active Ingredient	Package number	Number of DDDs	Number of PPI users*	Average package number per user per year	Average number of DDDs per user per year
A02BC01	omeprazole	225 518	6 817 868	54 739	4.12	124.55
A02BC02	pantoprazole	4 603 004	107 216 453	663 339	6.94	161.63
A02BC03	lansoprazole	417 055	10 595 214	57 440	7.26	184.46
A02BC04	rabeprazole	596 482	15 763 302	94 218	6.33	167.31
A02BC05	esomeprazole	1 282 824	39 386 331	226 502	5.66	173.89
All PPI (A02AB)		7 124 883	179 779 168	**1 096 238	6.50	164.00

*user: person who purchased a prescribed proton pump inhibitor product (of the given active ingredient) at least once a year.

**: estimation as sum of individual active agents users.

Due to generic competition, the previous decade brought a significant price reduction of PPIs, but still the National Health Insurance Fund of Hungary reimbursed 1622 million forints (5.1 million Euros) for these products in 2018 (**Table 4**).

**Table 4:** Reimbursement cost of proton pump inhibitors and reimbursement share of individual PPIs.

ATC	Active Ingredient	Reimbursement—million Hungarian forint—(%)				
		2014	2015	2016	2017	2018
A02BC01	omeprazole	401.45 (15.50)	349.41 (15.27)	44.00 (4.07)	40.93 (3.63)	98.29 (6.06)
A02BC02	pantoprazole	475.77 (18.37)	578.39 (25.28)	617.04 (57.06)	659.19 (58.42)	738.16 (45.51)
A02BC03	lansoprazole	241.72 (9.33)	190.65 (8.33)	81.47 (7.53)	76.07 (6.74)	97.11 (5.99)
A02BC04	rabeprazole	475.04 (18.34)	376.68 (16.46)	110.36 (10.21)	108.57 (9.62)	177.23 (10.93)
A02BC05	esomeprazole	996.07 (38.46)	793.06 (34.66)	228.55 (21.13)	243.58 (21.59)	511.16 (31.52)
All PPI (A02AB)		2590.04 (100.00)	2288.18 (100.00)	1081.43 (100.00)	1128.34 (100.00)	1621.95 (100.00)

### Patient-Level Surveys

The results are summarized in **Table 5** and **Table 6**. Among the hospitalized patients, the prevalence of PPI utilization was around 50% (ranged between 44.5% and 54.1% in different surveys), and all patients were on oral PPI therapy. Up to 21.5% of the patients had been put on PPI therapy during hospitalization (**Table 5**). However, in all surveys, most of the patients only continued their previously prescribed PPI treatment. PPI therapy was initiated by a general practitioner in 81.5% of cases, and in only a few cases was PPI treatment started by a gastroenterologist (Survey 3).

**Table 5:** Patient-level survey results.

	Survey 1	Survey 2	Survey 3
PPI users/all patients	382/706	399/864	65/146
prevalence (%)	54.11	46.18	44.52
95% confidence interval	50.42–57.75	42.88–49.51	36.70–52.62
Sex ratio (males per females)	183:199	192: 207	32:33
Males %	47.91	48.12	49.23
Age: mean ± SD	68.62 ± 15.11	69.59 ± 14.21	65.69 ± 11.56
min-max	18–96	23–98	31–86
Polypharmacy in the PPI user group (number of patients)	unknown	343	59
%		85.96	73.29
Administered active ingredient: number of patients (%)			
Pantoprazole	366 (95.81)	384 (96.24)	51 (78.46)
Lansoprazole	2 (0.52)	2 (0.5)	8 (12.31)
Rabeprazole	2 (0.52)	3 (0.75)	5 (7.69)
Omeprazole	3 (0.79)	2 (0.5)	0 (0.00)
esomeprazole	9 (2.36)	8 (2.01)	1 (1.54)
Duration of PPI treatment: number of patients (%)	unknown		
started during hospitalization/after admission		84 (21.05)	0 (0.00)
less than 1 year		99 (24.81)	3 (4.62)
1 to 5 years		125 (31.33)	48 (73.85)
more than 5 years		82 (20.55)	14 (21.54)
		missing answer: nine people	
Indication of PPI utilization: number of patients (%)			
Therapy			
GORD	48 (12.57)	41 (10.28)	8 (12.31)
Helicobacter eradication	6 (1.57)	1 (0.25)	4 (6.15)
Peptic ulcer long-term treatment	40 (10.47)	43 (10.78)	1 (1.54)
Prevention			
Stress ulcer prophylaxis	15 (3.93)	8 (2.01)	0 (0.00)
Other—”gastro protection” (e.g. ASA, NSAID, polypharmacy)	273 (71.47)	296 (74.19)	52 (80.00)
ASA	47 (12.3)	118 (29.57)	27 (41.54)
Clopidogrel	no info	48 (12.03)	42 (64.62)
ASA and Clopidogrel	no info	40 (10.03)	18 (27.69)
NSAID	64 (16.75)	52 (13.03)	3 (4.62)
Anticoagulants*	no info	no info	4 (6.15)
Missing answer	–	10 (2.51)	–

*Low molecular weight heparins, vitamin K antagonist, novel anticoagulants.

PPI, proton pump inhibitor; SD, standard deviation; GORD, Gastro-oesophageal reflux disease; ASA, acetyl salicylic acid; NSAID, non steroid anti-inflammatory drugs.

**Table 6:** Prescribed pantoprazole daily doses in patient-level surveys.

		Survey 1	Survey 2	Survey 3
	Number of patients (%)	N=366 (100)	N=384 (100)	N=51 (100)
**Pantoprazole daily dose**	20 mg (low dose/on demand dose)	51 (13.93)	72 (18.75)	12 (23.53)
	40 mg (standard dose-WHO DDD)	177 (48.36)	174 (45.31)	22 (43.14)
	60 mg	2 (0.55)		
	80 mg (high dose)	135 (36.80)	136 (35.42)	15 (29.41)
	120 mg			1 (1.96)
	160 mg		1 (0.26)	
	*missing data*	*1 (0.27)*	*1 (0.26)*	*1 (1.96)*

The average age of patients in all surveys was >65 years. The prevalence of polypharmacy among PPI users was >70%. In all surveys, the most commonly used active ingredient was pantoprazole. The prescribed daily dose of pantoprazole varied from 20 mg to 160 mg. According to our survey data, most patients received 40 mg (43.1%−48.4%) or 80 mg pantoprazole (29.4%–36.9%) per day (**Table 6**). Duration of PPI therapy was typically between 1 and 5 years. Nearly 20% of the patients had been taking PPIs for more than 5 years. None of the patients reported an attempt to de-prescribe (Survey 3).

In 20% of the cases, the indication for PPI utilization was therapy in all three surveys, mostly for gastro-esophageal reflux or peptic ulcer. However, most patients received PPI as prevention. Most patients had other diseases besides the main reason for their hospitalization, which is not surprising considering their average age. This means that most patients were on polypharmacy and PPIs were prescribed as prevention against potential gastric mucosal irritation caused by other medications (see indications in **Table 5**).

Applying a conservative estimate, in Survey 2, of the 399 PPI users, 66 patients (16.5%) did not have any therapeutic indication for PPI use, and also had not used any potentially ulcerogenic or anticoagulant/antiplatelet drug. Similarly, most patients in Survey 3 received clopidogrel therapy, but only a few of them had also received NSAID treatment, or had had a history of peptic ulcer or bleeding prior to hospitalization. Dual antiplatelet therapy administration occurred in up to 27.7% of the patients taking PPIs (**Table 5**).

## Discussion

Due to the superiority of PPIs over H2 receptor blockers in both effectiveness and pharmacokinetics, their use soon replaced H2 receptor blockers in the treatment of gastroenterological diseases caused by increased hydrochloric acid secretion [van Pinxteren et al., 2006; Kahrilas et al., 2008; National Institute for Health and Care Excellence (NICE), 2014; Iwakiri et al., 2016; Brunton, 2017; Savarino et al., 2017]. In addition, increased awareness of ulcer prophylaxis requirements has resulted in endless expansion of the PPI market (Savarino et al., 2017).

Besides official, evidence-based indications for PPI treatment and prophylaxis, unjustifiable prescriptions occurred in clinical practice (Savarino et al., 2017; Savarino et al., 2018a). A review found the mean rate of PPI overuse was 57% in hospitals, and 50% in primary care settings (Savarino et al., 2017).

### Prevalence of PPI Utilization

Based on national data, it was estimated that 14% of the Hungarian adult population was exposed to PPIs in outpatient care per year, while in adult hospital inpatients, this number was considerably higher, nearly 50%. Lower exposure was found in a Danish nationwide study, where gradual increase of PPI use was detected, and 7.4% of the adult population was exposed to PPIs in 2014. The high exposure of Hungarian patients to PPIs can be explained by the fact that PPI therapy can also be initiated by General practitioners (GPs) who can prescribe PPIs as gastroenterologists (i.e. with the same reimbursement rate). The high prevalence of PPI use (nearly 50% or above) among hospitalized patients has previously been reported in other countries as well (Chia et al., 2014; Kelly et al., 2015; Meli et al., 2015; Lodato et al., 2016; Pujal Herranz, 2016; Del Giorno et al., 2018).

### Active Ingredient/Pharmaceutical Form/Dosing of PPIs/Expenses

In Hungary (as in Denmark), pantoprazole was the most frequently prescribed and dispensed PPI, while in Iceland, omeprazole and esomeprazole were the most frequently used PPIs (Pottegard et al., 2016; Halfdanarson et al., 2018). The dominance of pantoprazole in Hungary can be explained by the high number of generic products and their consequent lower price compared to other PPI agents.

Every third patient in our study was on a high dose pantoprazole regimen, despite the fact that only those with extra-esophageal gastro-esophageal reflux symptoms (Zollinger–Elisson syndrome) require higher than standard dose PPI treatment (Savarino et al., 2018a). The high rate of high-dose pantoprazole treatment can be explained by the general suboptimal practice that despite the longer duration of action due to the irreversible inhibition of the proton pump, similarly to H_2_ receptor antagonists, doctors often prescribe PPI to be taken twice daily regardless of indication.

Moreover, in the most common indications for PPIs (symptomatic gastro-esophageal reflux, maintenance therapy of reflux esophagitis, or gastro protection with concomitant NSAID use), 20 mg pantoprazole (low dose) is recommended in the summary of product characteristics. In the Icelandic study, 95% of patients started high dose PPI (they defined this “high dose” category as standard dose or high dose), and 21% remained on that treatment after 1 year (Halfdanarson et al., 2018). Similar overdosing of PPIs has been reported from China (Ying et al., 2019).

Despite the continuous increase in PPI utilization in the study period, the reimbursement costs have decreased. The Hungarian reimbursement system is very complex and under continuous change (e.g. new prices are published monthly). During the five study years, the continuous growth in the number of available products (mainly pantoprazole) in the market generated a considerable price competition and lower reimbursement costs.

### Duration of PPI Use

Based on nationwide ambulatory drug use data, we estimated that each PPI user took PPIs for more than half a year on average. In the patient-level surveys, we observed that most patients were on PPI therapy for more than a year, and every fifth patient received PPI for more than 5 years. One possible explanation for the long duration of PPI treatment can be the lack of national guidelines on PPI use and PPI de-prescribing, and lack of medication review service provided by pharmacists. As the average age of PPI users admitted to the hospital was >65 years in all patient-level surveys, our data is similar to the findings of Halfdanarson et al., who reported remarkably higher PPI therapy duration in the elderly (Halfdanarson et al., 2018). Prolonged treatment was observed in other studies. In a Danish study, 44% of PPI users received PPI therapy for at least 3 years (Pottegard et al., 2016), while in Iceland 22% of PPI users remained on PPI treatment after 1 year (Halfdanarson et al., 2018). Appropriate duration of PPI use varies for different indications, but in general, it rarely exceeds 3 months in clinical guidelines (Halfdanarson et al., 2018). According to the newest National Institute for Health and Care Excellence guideline (National Institute for Health and Care Excellence (NICE), 2014), for the vast majority of patients with gastro-esophageal reflux [those without severe erosive esophagitis, Barrett’s Esophagus, or dilation of esophageal stricture) short-term PPI treatment is recommended for a maximum of 8 weeks (National Institute for Health and Care Excellence (NICE), 2014].

The estimated overall prevalence of gastro-esophageal reflux, the most common indication for PPI use, is around 9%–26% in the European population (El-Serag et al., 2014). The prevalence of diseases requiring long-term PPI use is low (Savarino et al., 2018a; Savarino et al., 2018b): e.g. severe erosive esophagitis (Los Angeles grade C/D) 0.5%, Barrett’s esophagus with confirmed intestinal metaplasia 1.6%, while Zollinger Ellison syndrome is a rare disease (Ronkainen et al., 2005a; Ronkainen et al., 2005b).

Considering continuous, long-term use of PPIs without the supervision of a gastroenterologist, duration of therapy is particularly alarming, since adverse events (e.g. *Clostridioies difficile* infection) may develop due to persistent PPI utilization, and the economic burden is also substantial (Bajor, 2017; Savarino et al., 2017; Ayele et al., 2018; Devitt et al., 2019).

### Initiation/De-Prescribing PPIs

Despite the fact that PPIs are available as Over the Counter products in Hungary, in none of the surveyed patients was the PPI therapy initiated by the patients themselves. According to Survey 3, which also assessed who initiated PPI use, most PPI regimens were started by GPs. In Iceland, GPs were also responsible for 60% of the PPI use (Halfdanarson et al., 2018). In Survey 2, which gathered data from 29 units, PPIs were initiated during the hospital stay in 20% of cases. Another US study evaluating PPI use at hospital admission and discharge found a higher rate of PPI continuation upon discharge (Gupta et al., 2010). As the prescribed drug regimen during the hospital stay is indicated on the discharge letter, this guides GPs to automatically continue prescribing PPIs. Pharmacist intervention could significantly promote rational PPI use in the hospital setting (Luo et al., 2018) by decreasing inappropriate indications, dosages and durations, so their medical reconciliation before discharge should be promoted to avoid irrational long-term PPI use.

According to the newest National Institute for Health and Care Excellence guideline [National Institute for Health and Care Excellence (NICE), 2014], periodic medication reviews are needed if PPI treatment is prolonged. The American guidelines also emphasize the need for de-prescribing, including using the lowest effective dose (Freedberg et al., 2017). De-prescribing guidelines are available for patients with uncomplicated, mild-moderate gastro-esophageal reflux disease who completed a minimum of 4 weeks of PPI therapy, and responded to it (Farrell et al., 2017; Freedberg et al., 2017). In our survey, none of the patients reported an attempt at de-prescribing. Automatic renewal of prescriptions without re-evaluation of patient symptoms is of great concern, as, without upper gastrointestinal endoscopy to confirm the presence of erosive esophagitis, long-term use of PPIs is debatable (Lassen et al., 2004). Also, ambulatory pH/impedance monitoring may help to distinguish gastro-esophageal reflux disease from a functional syndrome, and consequently avoid lifelong PPI therapy (Freedberg et al., 2017).

### Indications for PPI Use

Most patients in this study received PPI as a prophylactic agent. According to the literature, the most common drivers of PPI misuse are related to unjustified, long-term prophylactic use: prevention of gastro-duodenal ulcers in patients without risk factors for gastric injury (NSAID users, antiplatelet/anticoagulant therapy); stress ulcer prophylaxis in non-intensive care units; steroid therapy alone; selective serotonin reuptake inhibitor therapy alone (Pottegard et al., 2016; Savarino et al., 2018a). In the Icelandic study, nearly half of the patients used PPI concurrently with acetyl salicylic acid, NSAID, platelet inhibitors, or oral anticoagulant (Halfdanarson et al., 2018). The concurrent use of ulcerogenic agents and/or anticoagulant/antiplatelet drugs was also considerable in the PPI users in our study (see **Table 5**). On the other hand, in Survey 2, out of the 399 PPI users, 66 patients (16,5%) did not have any therapeutic indication for PPI use, and also had not used any potentially ulcerogenic or anticoagulant/antiplatelet drug, which is a clear evidence of PPI overuse. Similarly, in Survey 3 only a minority of patients taking clopidogrel received NSAID treatment, or had had a history of peptic ulcer/bleeding, or were on dual antiplatelet therapy, which may question the need for PPI use.

Other studies from inpatients reported a high discordance from evidence-based indications of PPI use (Pillans et al., 2000; Chia et al., 2014; Meli et al., 2015; Lodato et al., 2016; Pujal Herranz, 2016; van den Bemt et al., 2016; Ying et al., 2019). In the expert review of Savarino et al. PPIs are considered as “harmless, cheap remedy for any digestive problems by doctors” (Savarino et al., 2018a). Moreover, as a Chinese article revealed, procurement incentives for doctors may also play a role (Zeng et al., 2015) in the non-prudent prescribing of PPIs.

The present study has some limitations inherent in the database and the study design. The National Health Insurance Fund database contains drug dispensing data for only prescribed and reimbursed drugs. As over the counter PPI products and prescribed but non-reimbursed PPI products are not included in the database, the scale of nationwide use of PPIs has been underestimated. In the patient-level point prevalence survey the prevalence of PPI use was assessed on the given study days and from hospitals where daily drug dispensation is supervised by a pharmacist. This study design did not allow for the precise determination of hospital initiation of PPI use, which could occur during the hospitalisation but after the specific study day. As hospital PPI initiation could differ in hospitals without ward pharmacists, our results cannot be extrapolated to all Hungarian hospitals. On the other hand, this study focused on chronic PPI use, initiated before hospital admission. As we excluded specific gastroenterological units and did not apply any specific inclusion criteria for the inpatients, we believe that results of the multicenter surveys are generalizable for the hospitalized patients in Hungary. Also, much of the information (indication, duration, initiation, de-prescribing) were gained from the patients, so recall bias should be considered. In Survey 3, due to the low number of patients, the data on de-prescribing should be regarded as a signal, not as a valid, generalizable assessment. In the absence of national and local guidelines on PPI use, we did not aim to precisely determine the appropriateness of PPI therapy.

## Conclusion

Our data suggests that Hungarian patients may receive PPIs in high doses and for a long time. PPI use beyond the recommended indications were found, so PPI treatment should be initiated more cautiously for specific indications, and PPI dosing and duration of therapy should be reconsidered regularly.

## Data Availability Statement

The original contributions presented in the study are included in the article/**Supplementary Material**; further inquiries can be directed to the corresponding author.

## Ethics Statement

The studies involving human participants were reviewed and approved by Hungarian Medical Research Council. The patients/participants provided their written informed consent to participate in this study.

## Author Contributions

MM, RB, and GS had the original idea for the manuscript. MM, RB, MS, RV, and GS organized data collection. MM, RB, ZE, and GS contributed to the analysis. MM, RB, PD, KS, and RV drafted the manuscript, which was reviewed and approved by ZE, MS, and GS.

## Funding

The study was funded by the University of Szeged.

## Conflict of Interest

The authors declare that the research was conducted in the absence of any commercial or financial relationships that could be construed as a potential conflict of interest.
